# Validation of Brief Screening Tools to Identify Impaired Driving Among Older Adults in Australia

**DOI:** 10.1001/jamanetworkopen.2020.8263

**Published:** 2020-06-17

**Authors:** Kaarin J. Anstey, Ranmalee Eramudugolla, Md Hamidul Huque, Mark Horswill, Kim Kiely, Alex Black, Joanne Wood

**Affiliations:** 1 School of Psychology, University of New South Wales, Sydney, Australia; 2 Neuroscience Research Australia, Sydney, Australia; 3 UNSW Ageing Futures Institute, University of New South Wales, Sydney, Australia; 4 Centre for Vision and Eye Research, Institute of Health and Biomedical Innovation, Queensland University of Technology, Brisbane, Australia; 5 School of Psychology, University of Queensland, Brisbane, Australia

## Abstract

**Importance:**

There is an urgent need to develop evidence-based assessments to identify older individuals who may be unsafe drivers.

**Objective:**

To validate 8 off-road brief screening tests to predict on-road driving ability and to identify which combination of these provides the best prediction of older adults who will not pass an on-road driving test.

**Design, Setting, and Participants:**

This prognostic study was conducted between October 31, 2013, and May 10, 2017, using the criterion standard for screening tests, an on-road driving test, with analysis conducted from August 1, 2019, to April 2, 2020. A volunteer sample of older drivers was recruited from community advertisements, rehabilitation and driver assessment clinics, and an optometry clinic in Canberra and Brisbane, Australia.

**Exposures:**

Off-road driver screening measures, including the Useful Field of View, DriveSafe/DriveAware, Multi-D battery, Trails B, Maze test, Hazard Perception Test, DriveSafe Intersection test, and 14-item Road Law test.

**Main Outcomes and Measures:**

Classification as unsafe on a standardized 50-minute on-road driving assessment administered by a driving instructor and an occupational therapist masked to the participant’s clinical diagnosis and off-road test performance.

**Results:**

A total of 560 drivers aged 63 to 94 years (mean [SD] age, 74.7 [6.2] years]; 350 [62.5%] men) were assessed. Logistic regression and receiver operating characteristic analyses indicated the area under the curve was largest for a multivariate model comprising the Multi-D, Useful Field of View, and Hazard Perception Test, with an area under the curve of 0.89 (95% CI, 0.85-0.94), sensitivity of 80.4%, and specificity of 84.1% for predicting unsafe drivers. The Multi-D battery was the most accurate individual assessment and had an area under the curve of 0.85 (95% CI, 0.79-0.90), sensitivity of 77.1%, and specificity of 82.1%. The multivariate model had sensitivity of 83.3% and specificity of 91.8% in the cognitively impaired group and sensitivity of 87.5% and specificity of 70.8% in the visually impaired group.

**Conclusions and Relevance:**

These findings suggest that off-road screening tests can reliably identify older drivers with a strong probability of failing an on-road driving test. Implementation of these measures could enable better targeting of resources for managing older driver licensing and support injury prevention strategies in this group.

## Key Points

**Question:**

Are brief off-road screening measures sufficiently sensitive and specific to identify older drivers who will not pass an on-road driving test in Australia?

**Findings:**

This prognostic study including 560 drivers aged 63 years and older found that off-road screening tests could identify older drivers who would not pass an on-road driving test with 77% sensitivity and 82% specificity. A combination of measures drawing from multiple skill domains provided the best prediction.

**Meaning:**

These findings suggest that brief off-road screening tests could be a cost-effective, objective tool to screen older drivers to determine who might be an unsafe driver and to indicate referral for an on-road driving test.

## Teaser

This prognostic study examines participants’ scores on 8 screening tools to identify drivers who are potentially unsafe compared with on-road driving test scores.

## Introduction

While road safety overall is increasing with safer vehicles, ongoing demographic changes have led to sharp increases in the prevalence of road trauma involving older drivers. In Australia, road users aged 65 years and older accounted for 14.9% of road deaths in 2009, increasing to 21.3% in 2018.^1^ Hospitalizations for injuries have also increased at a higher rate among older road users than younger road users, with similar statistics reported for the US.^1,2^ Cognitive impairment and visual impairment are the 2 functional domains that have the largest effect on safety among older drivers and arguably pose the greatest difficulty for clinicians in terms of evaluation of driving risk.^3,4,5,6,7^ An on-road driving test is the criterion standard for evaluating the safety of an older driver but can be expensive and time consuming. Screening tools of driver skills validated against an on-road test provide an urgently needed alternative.

### Appropriate Methods to Evaluate Validity of Screening Measures

Several off-road screening tests have been developed to assist with clinical decision-making for older drivers in the US and Australia.^8,9,10,11,12^ However, limited data are available on the validity of most of these tests, and to our knowledge, they have never been compared within the same study on the same group of participants who have been assessed using the same on-road test. Our study addresses this gap in the literature, with a large sample of older drivers who underwent an on-road driving test.

### Study Aims

We aimed to prospectively validate 8 brief measures used clinically or in research settings to identify drivers who would be classified as unsafe using an on-road driving assessment. Analysis of all measures in the single sample allows their relative predictive accuracy to be evaluated. We also sought to identify the optimal combinations of these measures to provide reliable off-road information for clinicians. We evaluated these measures in subgroups of participants with cognitive and visual impairment.

## Methods

The study protocol was approved by the Human Research Ethics Committees of the Australian National University, Australian Capital Territory Government Health Directorate, and Queensland University of Technology. Written informed consent was obtained from all participants. This study is reported following the Standards for Reporting of Diagnostic Accuracy (STARD) reporting guideline (eTable 1 in the Supplement).

### Study Population

The Driving Aging Safety and Health project was undertaken in the Australian cities of Canberra and Brisbane. In Canberra, community dwelling drivers aged 65 years and over with a current valid license were recruited through advertisements in newspapers, community groups, and general practice and health clinics between October 31, 2013, and May 10, 2017. They were also recruited into the sample from the Australian Capital Territory Health Disability and Rehabilitation Service (DARS), which assesses drivers who are medically fit to drive but referred owing to concerns about driving safety, often in relation to cognitive impairment. Clients of DARS whose licenses were revoked were excluded from participation. Participants in Canberra completed a neuropsychological battery to identify mild cognitive impairment (MCI), as defined by Anstey et al.^13^ Participants in Brisbane aged 60 years and older with eye disease were recruited through the Queensland University of Technology Optometry Health Clinic and completed a full eye examination, including central and peripheral visual function testing.

Prevalence of unsafe driving was expected to be approximately 30.0% in older drivers with MCI,^14^ 35.0% in older drivers who were visually impaired,^15^ and 20.0% in the general population of older drivers.^12^ Study sample size was planned assuming the sensitivity of Multi-D^12^ to detect unsafe driving to be between 80.0% and 91.0%. Assuming 80% power and 5% critical error, a minimum of 67 drivers who would be considered unsafe were required per group; therefore, we planned required total samples of 223 participants with MCI, 191 participants who were visually impaired, and 335 participants without clinical impairment.

 Recruitment via the electoral role was planned to obtain a representative sample of older drivers for the comparison groups but was not possible owing to prevailing policies that classified the study as nonmedical.

### Procedure and Measures

Assessments were conducted at either the Australian National University in Canberra or Queensland University of Technology in Brisbane. Questionnaires on driving habits, medical conditions, falls, and instrumental activities of daily living were administered.^16^ Off-road driver screening assessments were conducted by a trained research assistant prior to the participant undergoing an on-road driving test. A standardized research on-road driving test was administered by a driver-trained occupational therapist and qualified driving instructor masked to the results of the off-road driver screening assessments (described elsewhere^13^). The outcomes of the off-road screening assessments were not available to those conducting the on-road test.

### Off-Road Screening Tests for Driving Safety

Eight different tests that have been reported in the literature to screen for unsafe driving or crashes in older adults were included in the laboratory assessment: (1) Trail Making Test B from the Halstead-Reitan Battery of neuropsychological tests^17^; (2) the Useful Field of View (UFOV) subtest 2, a computer-based test of visual processing speed and divided attention with high reliability and validity demonstrated in other large studies to predict crash risk^10,18^; (3) DriveSafe/DriveAware,^9^ a computer-based commercially available clinical screening tool for driving safety^19^ (the DriveSafe component of this test was used, which requires participants to view each of 15 images of the same roundabout intersection for 3 seconds, then verbally report details about the position and direction of travel of pedestrians and vehicle in the image)^9^; (4) DriveSafe Intersections, an additional multiple-choice questionnaire that assessed knowledge of road law; (5) the Hazard Perception Test (HPT),^20^ a computer-based test requiring participants to view each of 20 video clips of 15 to 40 seconds’ duration depicting a traffic conflict and respond via touchscreen when they observed a potential traffic hazard in the video, which has been previously validated against self-reported crashes in older adults^8^; (6) the 14-item Road Rules and Road Craft test (Road Law), which comprises 14 questions about road safety and has been correlated with on-road test performance in clinical and research samples^11^; (7) the Multi-D, a computer-based test that includes 3 subtests (choice color reaction time [requiring hand and foot responses and inhibition of responses], a test of sensitivity to central visual motion [using random dot stimuli presented at 3.2 m], and a test of balance or postural sway [using a sway meter to measure body displacement at the level of the waist]) and has been associated with on-road driving performance^12,21^; and (8) the Snellgrove Maze test,^22^ a pen-and-paper maze test for which time taken to complete the maze has been associated with driving performance.^22,23^

### On-Road Driving Test

At each site, within 3 months of the off-road assessment, participants completed a 50-minute open road assessment in an automatic vehicle with dual brake controls fitted. This followed a standard, validated protocol and scoring procedure that has been described previously.^24,25^ Drivers’ safety was rated by an occupational therapist on a scale of 1 to 10, with a score of 1 to 3 indicating serious errors, unsafe driving behavior, and low likelihood of passing a driving test, while a score between 4 and 10 indicated a higher level of safety and likelihood of passing a driving test. Per our previous research, the occupational therapist and driving instructor were not involved in the administration of the off-road screening tests and were not informed of any results from these tests and hence were masked.^12,13,26^ Interrater reliability of the safety rating was high (intraclass correlation = 0.94 [95% CI, 0.93 to 0.95]). Further details are provided in the eAppendix in the Supplement.

### Classification of Cognitive Impairment and Eye Disease

In Canberra, participants were included in the cognitive impairment subgroup if they were reviewed by the DARS clinic, had a known dementia diagnosis, had an Mini-Mental State Examination score less than 23, or met general criteria for MCI. A validated psychometric approach was used to classify participants^27^ with MCI (eAppendix and eTable 2 in the Supplement).^28^ Participants were classified as having MCI if they demonstrated subjective memory concern (Memory Complaints Questionnaire MAC-Q score >24), mild impairment (between 1 to 2 SDs below reference ranges) on 1 or more of 6 neurocognitive domains based on a battery of 11 cognitive measures (eTable 2 and eTable 3 in the Supplement), no or minimal self-reported impairment of instrumental activities of daily living due to cognition, and no evidence of dementia.^13^

 In Brisbane, participants from any of the recruitment sources were classified as having visual impairment from any form of eye disease if they demonstrated any combination of central vision loss (assessed by visual acuity and/or contrast sensitivity), peripheral vision loss (defined as the presence of any significant visual field defects in either eye as defined by automated perimetry), or confirmed diagnosis by an eye care practitioner or evidence of ocular disease detected during the eye examination.

### Statistical Analysis

To conduct cross-sectional validation and comparison of the off-road screening measures against on-road safety, we used logistic regression to evaluate each measure against the on-road safety rating (safe: rating >3 vs unsafe: rating ≤3) adjusting for site (Canberra or Brisbane). We used this logistic regression to obtain a receiver operating characteristics (ROC) analysis of each measure. We obtained optimal cutoff points based on the Youden Index to estimate sensitivity, specificity, positive predictive value (PPV), negative predictive value (NPV), and accuracy of each screening measures based on this cutoff. We repeated these analyses on subgroups with eye disease and MCI to evaluate the validity of the screening tests for these clinical populations. Finally, we used multivariable logistic regression with backward elimination (*P* < .05) to evaluate the combination of best performing screening measures against on-road driving test outcome, adjusting for the data collection site. To examine the predictability of the best performing screening measures combinations, they were further assessed using leave-one-out and 10-fold cross validation. To address missing data in covariates, we carried out sensitivity analysis using a fully-conditional specification^29^ technique. Twenty imputed data sets were considered, and estimates from these data sets were later combined to obtain the pooled estimates with the Rubin rule. Analyses were conducted using Stata statistical software version 16.0 (StataCorp). *P* values were 2-sided, and statistical significance was set at *P* < .05. Data were analyzed from August 1, 2019, to April 2, 2020.

## Results

### Sample Characteristics

**Figure 1.**
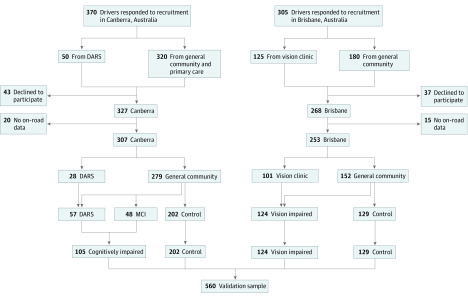
Study Sample Flowchart DARS indicates Disability and Rehabilitation Service; MCI, mild cognitive impairment.

Of 675 individuals who responded to recruitment, 560 participated in the study and also completed the on-road driving test (Figure 1). The age range was 63 to 94 years (mean [SD], 74.7 [6.2] years; 350 [62.5%] men). Participants had a median (interquartile range [IQR]) of 15 (2-12) years of education, and 68 participants (12.1%) were classified as unsafe drivers according to the on-road test. In the Canberra sample, 105 participants were classified as having cognitive impairment (either met MCI criteria or were referred to DARS), and in the Brisbane sample, 124 participants had clinically significant visual impairment. Participants in the comparison group from the Canberra site, compared with those from the Brisbane site, were older (median [IQR] age, 75.0 [71.0 to 80.0] years vs 71.0 [68.0 to 76.0] years), had more years of education (median [IQR], 16.0 [13.0 to 18.0] years vs 14.0 [12.0 to 17.0] years), performed worse on Road Law (median [IQR] score, 32.0 [30.0 to 34.0] vs 35.0 [33.0 to 36.0]), Multi-D (median [IQR] score, −4.44 [−5.27 to −3.47] vs −4.25 [−5.24 to −3.21]), visual acuity (median [IQR] score, 0 [−0.1 to 0.1] vs −0.1 [−0.1 to 0]) and contrast sensitivity (median [IQR] score, 1.7 [1.7 to 1.7] vs 1.8 [1.8 to 1.8]), and on-road test (18 drivers who were unsafe [8.7%] vs 9 drivers who were unsafe [7.0%]), and better on UFOV (median [IQR] score, 83.0 [30.0 to 167.0] vs 97.0 [43.0 to 222.0]), and HPT (median [IQR], 5.5 [4.8 to 6.5] seconds vs 6.1 [5.2 to 7.0] seconds) (Table 1). There were more men in the cognitively impaired (66 [62.9%] men) and visually impaired (87 [70.2%] men) groups than in the comparison groups in Canberra (119 men [58.9%]) and Brisbane (78 [60.5%] men).

**Table 1.** Sample Characteristics by Site and Subgroup

Characteristic	Group, No. (%)			
	Canberra (n = 307)		Brisbane (n = 253)	
	Comparison (n = 202)	Cognitively impaired (n = 105)	Comparison (n = 129)	Vision impaired (n = 124)
Age, median (IQR), y	75.0 (71.0 to 80.0)	77.0 (71.0 to 80.0)	71.0 (68.0 to 76.0)	74.0 (70.0 to 78.0)
Men	119 (58.9)	66 (62.9)	78 (60.5)	87 (70.2)
Education, median (IQR), y	16.0 (13.0 to 18.0)	16.0 (12.0 to 19.0)	14.0 (12.0 to 17.0)	14.0 (11.0 to 17.0)
MMSE, median (IQR)	29.0 (28.0 to 30.0)	29.0 (28.0 to 30.0)	29.0 (28.0 to 30.0)	29.0 (28.0 to 30.0)
Screening Test score, median (IQR)				
UFOV, ms^a^	83.0 (30.0 to 167.0)	137.0 (40.0 to 280.0)	97.0 (43.0 to 222.0)	153.0 (77.0 to 263.0)
DriveSafe^b^	92.0 (82.0 to 100.0)	84.0 (73.0 to 96.0)	94.0 (78.0 to 107.0)	86.0 (70.0 to 100.0)
DriveSafe Intersection^b^	7.0 (6.0 to 7.0)	6.0 (5.0 to 7.0)	7 (6.0 to 7.5)	6.0 (5.0 to 7.0)
14-Item Road Law^b^	32.0 (30.0 to 34.0)	32.0 (29.0 to 34.0)	35.0 (33.0 to 36.0)	35.0 (33.0 to 36.0)
Maze test time, s^a^	23.9 (17.0 to 32.0)	28.2 (21.0 to 37.0)	24 (17.4 to 30.8)	25.5 (19.7 to 33.3)
TMT-B, s^a^	96.5 (74.4 to 124.3)	123.0 (89.0 to 177.8)	96.7 (83.5 to 131.9)	111.4 (87.3 to 148.8)
HPT response time, s^a^	5.5 (4.8 to 6.5)	6.2 (5.0 to 7.6)	6.1 (5.2 to 7.0)	6.2 (5.3 to 7.6)
Multi-D, median (IQR)				
Choice reaction time, s^a^	0.9 (0.8 to 1.0)	0.9 (0.8 to 1.1)	0.9 (0.8 to 1.0)	0.9 (0.8 to 1.0)
Dot motion (logDegArc)^b^	−1.8(−2.0 to −1.7)	−1.8 (−1.9 to −1.6)	−1.8 (−1.9 to −1.7)	−1.6 (−1.8 to −1.5)
Sway path length, mm^a^	141.0 (114.0 to 192.0)	163.5 (120.5 to 235.5)	121.0 (89.5 to 148.0)	132.5 (110.0 to 177.5)
Vision, median (IQR)^a^				
High contrast acuity both eyes, logMAR	0 (−0.1 to 0.1)	0 (0 to 0.1)	−0.1 (−0.1 to 0.0)	0 (−0.1 to 0.1)
Contrast sensitivity, logCS	1.7 (1.7 to 1.7)	1.7 (1.6 to 1.7)	1.8 (1.8 to 1.8)	1.8 (1.7 to 1.8)
Unsafe score in on-road test	18 (8.7)	13 (10.9)	9 (7.0)	25 (20.2)
Driving habits				
Distance/wk, km				
<50	22 (10.9)	19 (18.1)	27 (20.9)	20 (16.1)
51-100	35 (17.3)	27 (25.7)	29 (22.5)	35 (28.2)
101-200	76 (37.6)	30 (28.6)	36 (27.9)	35 (28.2)
>200	56 (27.7)	24 (22.9)	33 (25.6)	31 (25.0)
Missing	13 (6.4)	5 (4.8)	4 (3.1)	3 (2.4)
Frequency, d/wk				
<3	6 (3.0)	10 (9.5)	7 (5.4)	9 (7.3)
3-4	35 (17.3)	18 (17.1)	27 (20.9)	31 (25.0)
5-6	75 (37.1)	35 (33.3)	51 (39.5)	44 (35.5)
Every day	80 (39.6)	40 (38.1	41 (31.8)	40 (32.3)
Missing	6 (3.0)	2 (1.9)	3 (2.3)	0
Motor vehicle collisions				
Past 12 mo				
0	171 (84.7)	83 (79.0)	115 (89.1)	112 (90.3)
1	20 (9.9)	18 (17.1)	13 (10.1)	12 (9.7)
2	2 (1.0)	0	0	0
Missing	9 (4.5)	4 (3.8)	1 (0.8)	0
Past 5 y				
0	140 (69.3)	69 (65.7)	93 (72.1)	90 (72.6)
1	39 (19.3)	25 (23.8)	25 (19.4)	26 (21)
2	10 (5.0)	4 (3.8)	8 (6.2)	5 (4)
3	2 (1.0)	3 (2.9)	2 (1.6)	2 (1.6)
4	0	0	0	1 (0.8)
Missing	11 (5.4)	4 (3.8)	1 (0.8)	0

Abbreviations: HPT, Hazard Perception Test; IQR, interquartile range; MMSE, Mini Mental State Examination; TMT-B, Trail Making Test Part B; UFOV, Useful Field of View.

^a^ Lower values represent better performance.

^b^ Higher values represent better performance.

In terms of adverse events, at the Canberra site, 1 participant (0.3%) nearly fell during the postural sway test and 3 participants (1.0%) had their on-road tests stopped early owing to safety concerns, but there were no injuries or traffic collisions. At the Brisbane site, 17 participants (6.7%) had their on-road test stopped early, and there were no injuries or traffic collisions.

### Cross-Sectional Analyses of Screening Measures as Predictors of On-Road Test Safety

The 10-point on-road driving test safety rating was dichotomized (unsafe score: ≤3; safe score: >3) and used as the outcome measure in logistic regression models that estimated individual associations of screening measures with driving safety (Table 2). All measures predicted on-road driving test performance in complete case analysis and in the multiply imputed data set in the combined data set of 560 drivers. Sensitivity, specificity, PPV, NPV, and the percentage correctly classified are reported in Table 3. No test had high PPV, but they all had high NPVs (ie, >90.0%), suggesting that these tests correctly classify safe drivers as safe. Overall statistics were best for the Multi-D in these univariate analyses (sensitivity, 77.1%; specificity, 82.1%; PPV, 33.3%; NPV, 96.9%; 81.6% of participants correctly classified).

**Table 2.** Logistic Regression Coefficients for Each Screening Measure as Predictors of On-Road Test Safety Category

Screening measure	On-road safety^a^					
	Complete case analysis			Multiple imputation		
	OR (95% CI)	*P* value	AUC	OR (95% CI)	*P* value	AUC
Trail Making Test B	1.01 (1.01-1.02)	<.001	0.75	1.01 (1.01-1.02)	<.001	0.75
Useful Field of View	1.01 (1.00-1.01)	<.001	0.76	1.01 (1.01-1.01)	<.001	0.76
DriveSafe	0.95 (0.93-0.96)	<.001	0.76	0.95 (0.93-0.96)	<.001	0.76
DriveSafe Intersection test	0.70 (0.58-0.84)	<.001	0.67	0.68 (0.57-0.82)	<.001	0.68
Hazard perception Response Time	1.70 (1.43-2.01)	<.001	0.72	1.69 (1.42-2.00)	<.001	0.72
14-Item Road Law Test	0.91 (0.86-0.97)	.002	0.62	0.90 (0.85-0.96)	.001	0.63
Multi-D Battery	2.64 (2.00-3.49)	<.001	0.85	2.51 (1.98-3.17)	<.001	0.84
Maze Test	1.03 (1.02-1.05)	<.001	0.65	1.03 (1.02-1.05)	<.001	0.68

Abbrevations: AUC, area under the curve; OR, odds ratio.

^a^ Using safe driving as the reference value. All models were adjusted for data collection site.

**Table 3.** Diagnostic Characteristics of Each of the Screening Test Under Respective Optimal Cut-Offs

Screening measure	%^a^				
	Sensitivity	Specificity	PPV	NPV	Correctly classified
Useful Field of View	50.8	85.7	32.7	92.7	81.5
DriveSafe	59.4	78.8	27.3	93.5	76.5
Maze Test	67.2	60.9	19.1	93.1	61.7
Trail Making Test B	75.4	63.9	21.9	95.1	65.3
Hazard perception response time	75.4	57.4	19.4	94.5	59.6
Multi-D Battery	77.1	82.1	33.3	96.9	81.6
DriveSafe Intersection test	66.7	54.4	15.6	92.8	55.8
14-Item Road Law Test	29.5	89.0	25.7	90.7	82.1

Abbreviations: NPV, negative predictive value; PPV, positive predictive value.

^a^ Analyses adjusted for site; PPV and NPV are highly dependent on the prevalence of unsafe driving in the sample.

### ROC Analyses

The dichotomized on-road driving test safety rating was used as the reference standard in ROC analyses for each screening measure. Table 2 displays the AUC for each measure adjusted for site. The AUCs ranged from 0.62 to 0.85 in the complete case analysis, with Multi-D battery test scoring highest (AUC, 0.85 [95% CI, 0.79 to 0.90]) among the measures compared.

**Figure 2.**
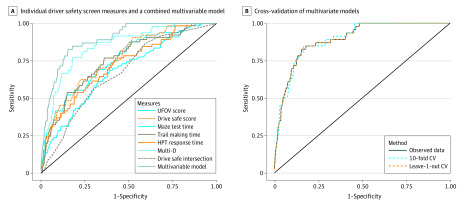
Receiver Operating Characteristic (ROC) Curve Showing Sensitivity and Specificity CV indicates cross-validation.

Multivariate analyses found that a combination of Multi-D, UFOV, and HPT remained significant when all variables were tested simultaneously in the model in complete case analysis, with an AUC of 0.89 (95% CI, 0.85 to 0.94) (eTable 4 in the Supplement). This model provides an estimated sensitivity of 80.4% and specificity of 84.1%. We obtained a similar AUC from the multiple imputed data sets with same covariates (eTable 4 in the Supplement). Figure 2A shows the ROC curve for the multivariate model, indicating a significant improvement in classification when combining measures. Both 10-fold and leave-one-out cross-validation analyses resulted in very similar AUC estimates for the combination of Multi-D, UFOV, and HPT to the complete case analysis (Figure 2B).

### Subgroup Analysis for Visual and Cognitive Impairment

Results for subgroups are shown in eTable 5 in the Supplement, including visually impaired, cognitively impaired, and the comparison group at each site. In the visually impaired group, all measures except the 14-Item Road Law Test and DriveSafe Intersection Test predicted safe driving on the on-road test. In the cognitively impaired group, the Maze Test, UFOV, and DriveSafe Intersection Test did not identify unsafe drivers. Sensitivity, specificity, PPV, and NPV by subgroup are shown in eTable 6 in the Supplement. The multivariate battery provided the highest sensitivity and specificity in the subgroups (eTable 6 in the Supplement). The multivariate model had 83.3% and 91.8% in the cognitively impaired group and 87.5% sensitivity 70.8% specificity in the visually impaired group.

## Discussion

This prognostic study found that reliable classification of drivers who are unsafe with an off-road screening assessment is possible. Of the individual tests evaluated, the Multi-D, HPT, and UFOV performed well. A combination of these tests provided the most robust assessment of driving safety off-road and could be suitable for clinical groups with visual impairment or cognitive impairment. This is likely to be because a range of measures is needed to identify impairments or weaknesses in the skills and abilities underlying safe driving performance. The Multi-D assessment provided the best prediction of any individual test across subsamples and the combined sample.

A low PPV and high NPV is to be expected for tests for rare conditions, as both statistics are influenced by prevalence. Our 12.1% rate of unsafe driving resulted in a low PPV, although sensitivity and specificity were high. Nevertheless, the rate of unsafe drivers in our sample is comparable to that reported in prior studies that included samples from the general population, such as 5.4% reported by Vaucher et al,^30^ 16.7% reported by Classen et al,^31^ or 17.4% reported by Wood et al,^12^ but lower than the prevalence in samples sourced only through clinics, such as 31.0% reported by Niewoehner et al among veterans referred to a driving clinic^15^ and by Kay et al among participants recruited from rehabilitation facilities,^19^ and 65.7% reported by Carr et al among drivers with dementia.^22^ The high sensitivity and specificity for the multivariate model indicate that the PPV would be slightly better than what we observed in our data if the model was used with a sample containing a higher prevalence of drivers who were unsafe.

The results of this study address a critical gap in the evidence on older driver assessment. Our findings demonstrate that it is possible to screen older drivers using off-road tests. This is highly significant because this approach would save the cost and time of an on-road driving assessment for many drivers. With populations aging, many countries lack sufficient resources to provide on-road driving assessments. The use of an objective assessment battery relieves physicians of the need to make subjective or clinical judgements about fitness to drive. We have previously shown that driver screening measures perform better than neuropsychological assessments for identifying unsafe driving.^13^

Further research is required to evaluate the frequency at which older drivers require assessment and to identify cutoffs for safety in specific driving populations. Quantification of crash risk associated with different levels of performance could be possible if large, prospective studies were conducted with these screening tests measured at baseline. Future research could also provide health economic evaluations of screening tools for older drivers.

### Strengths and Limitations

Our project addressed several limitations of prior studies. To our knowledge, this is the first study to evaluate several off-road screening tests on the same samples, allowing us to compare the utility of each test for identifying drivers who may be unsafe or safe. We included a larger sample with on-road test results than has been studied previously for this purpose, to our knowledge.^32^ Most prior studies have included fewer than 200 participants, with the previous largest study including 425 participants.^33^ Our larger sample provides more precise estimates of the accuracy of these tests in predicting unsafe driving behavior in older adults. Previously, reviews^34,35^ have attempted to compare the statistical properties of tests despite tests being evaluated on studies that differ in the prevalence rate of unsafe driving, sample characteristics, and clinical status. In prior research, typically only 1 or 2 tests were evaluated. Our on-road driving test assessors were blind to results of the screening tests, and we evaluated both imputed data sets and complete cases. We also evaluated the screening tests on 2 preclinical groups of drivers who are at highest risk of becoming unsafe on the road and are regularly treated by physicians.

This study also has some limitations, including our use of nonrandom sampling. Participants were recruited via referral and advertisements; therefore, they were not necessarily representative of the older driver population, as they are subject to selection effects. However, this limitation is common to most driving research and also occurs in clinical samples. While on-road tests remain the criterion standard for determining unsafe driving performance, they do not assess performance in all driving situations to which drivers are typically exposed. We did not link our results to state crash records because we have previously shown that these have low accuracy in Australia.^36^ However further evaluation of these measures in relation to actual crash data, as well as naturalistic driving is warranted.

## Conclusions

The findings of this prognostic study suggest that there is sufficient evidence to use off-road screening tests to identify older drivers who may be unsafe. Validated off-road tests are cost effective and efficient, and they could be a more cost-effective approach to manage safety among older drivers.

## Notes

Supplement. **eAppendix.** Supplementary Methods **eTable 1.** STARD Checklist **eTable 2.** Criteria and Measures for Psychometric Classification of Mild Cognitive Impairment **eTable 3.** Performance on Age, Sex, and Education Standardized Domain Measures of Neurocognitive Function for the Canberra Sample by Subgroup **eTable 4.** Multivariate Logistic Regression Coefficients for Combination of Factors Associated With On-Road Test Safety **eTable 5.** Logistic Regression **eTable 6.** Sensitivity and Specificity Across All Subgroups
