# Error in Methods

**DOI:** 10.1001/jamanetworkopen.2020.20705

**Published:** 2020-08-31

In the Original Investigation titled “Validation of Brief Screening Tools to Identify Impaired Driving Among Older Adults in Australia,”^1^ published June 17, 2020, there was an error in the Methods in the description of the maze test used. The correct maze test is Occupational Therapy—Driver Off Road Assessment maze test. This article has been corrected.^1^
