# Supplementary material for: Validation of Brief Screening Tools to Identify Impaired Driving Among Older Adults in Australia
Source: JAMA Netw Open. 2020 Jun 17;3(6):e208263. doi: 10.1001/jamanetworkopen.2020.8263 (PMC7301240; doi:10.1001/jamanetworkopen.2020.8263)
Supplement: Supplement. — eAppendix. Supplementary Methods eTable 1. STARD Checklist eTable 2. Criteria and Measures for Psychometric Classification of Mild Cognitive Impairment eTable 3. Performance on Age, Sex, and Education Standardized Domain Measures of Neurocognitive Function for the Canberra Sample by Subgroup eTable 4. Multivariate Logistic Regression Coefficients for Combination of Factors Associated With On-Road Test Safety eTable 5. Logistic Regression eTable 6. Sensitivity and Specificity Across All Subgroups [file jamanetwopen-3-e208263-s001.pdf]

Supplementary Online Content

Anstey KJ, Eramudugolla R, Hugue MH, et al. Validation of brief screening tools to identify impaired driving among older adults in Australia. *JAMA Netw Open*. 2020;3(6):e208263. doi:10.1001/jamanetworkopen.2020.8263

eAppendix. Supplementary Methods

eTable 1. STARD Checklist

eTable 2. Criteria and Measures for Psychometric Classification of Mild Cognitive Impairment

eTable 3. Performance on Age, Sex, and Education Standardized Domain Measures of Neurocognitive Function for the Canberra Sample by Subgroup

eTable 4. Multivariate Logistic Regression Coefficients for Combination of Factors Associated With On-Road Test Safety

eTable 5. Logistic Regression

eTable 6. Sensitivity and Specificity Across All Subgroups

This supplementary material has been provided by the authors to give readers additional information about their work.

© 2020 Anstey KJ et al. *JAMA Network Open.*

eAppendix. Supplementary Methods

Section 1. Classification of Cognitive Impairment

Participants at the Canberra site took part in a detailed cognitive assessment battery conducted in person by a trained research assistant. The standardized cognitive tests included Mini-Mental Status Exam (MMSE), Victoria Stroop Test (Part A: dots, Part B: non-colour words; Part C: colour-words), California Verbal Learning Test (CVLT) immediate recall and delayed recall, Controlled Oral Word Association Test (COWAT), Boston Naming Test 15-item, Benton Visual Retention Test Copy, Trail Making Test Part B, Wechsler Digit Span Backwards, and the Game of Dice Test. Participants also completed in survey format, a validated memory concerns questionnaire (Memory Complaints Questionnaire MAC-Q) and questions regarding difficulty with instrumental activities of daily living (shopping, meal preparation, using a map, making telephone calls, or taking medications), including whether those difficulties were due to memory issues or physical health issues, or both.

An algorithm was used to classify participants as meeting International Working Group (IWG) General criteria for MCI. These criteria were: absence of dementia, presence of subjective cognitive decline, presence of objective cognitive impairment on testing, and minimal impairment of IADLs. MCI etiology was not considered because this was beyond the scope of the present study, and the target population of older drivers presenting to primary care physicians for Fitness to Drive assessment is typically etiologically heterogeneous. The IWG criteria (rather than other more recent diagnostic frameworks such as DSM-5 mild neurocognitive disorder) was selected because MCI is currently the most commonly used diagnostic definition among clinicians for a pre-clinical dementia stage (1, 2).

The algorithm evaluated participant testing and survey data against each of the general MCI criteria using validated cut-off scores as presented in eTable 2. This approach has been previously validated against expert diagnosis in a sample of 1644 Australian adults aged 72-76 years(3). Participants' performance on each of the neurocognitive domains is presented in eTable 3. In general, participants in the Cognitively Impaired sub-group as a whole (which included participants meeting criteria for either MCI or dementia, and those referred to the Driver Assessment and Rehabilitation Clinic without MCI or dementia), demonstrated cognitive performance approximately 0.5 standard deviations below that of the Comparison Group.

Section 2. On-Road Driving Test Method

The route was pre-determined and incorporated situations drivers typically encounter during suburban driving. All assessments were conducted during daylight, non-peak traffic hours. Although the driving context, traffic density and roads are different between the two cities, the standardized routes were carefully mapped to be of similar duration (45-50 minutes at both sites) and distance (19-20 km at both sites), and to include similar components. At each site, route components included: traffic light controlled intersections, non-traffic controlled intersections (i.e., stop signs, give way signs), roundabouts, straight driving along single carriage as well as dual carriage roads, curved driving along single and dual carriage roads, highway driving (80-100km/hr zones), residential area driving (50-60km/hr), active school zones (Canberra only), pedestrian crossings, chicanes, one-way roads (Brisbane only),

© 2020 Anstey KJ et al. *JAMA Network Open.*

parking, 20 meter reverse, three-point turn and pull-in pull-out maneuver. At both sites, the driving instructor provided navigation instructions for 80% of the driving route. The remaining 20% of the drive was completed under self-navigation conditions where participants were instructed to drive to a pre-determined destination.

The scoring protocol was adapted from the methods typically used by driver trained Occupational Therapists (OT) in Australia when conducting on-road assessments. Seated in the rear passenger seat, the OT scored the participants' driving performance in the areas of general observation (scanning and attention), blind spot checks, lane positioning, braking/acceleration (appropriate speed and braking), gap selection (gap selected when entering traffic or the gap between the driver and other vehicles) and approach to hazards (appropriate planning and preparation).(4) Indication/signaling (appropriate use of directional indicator) was also assessed where appropriate. The final driver safety rating was standardized by ensuring OTs at both sites used a 1-10 scale at each site. Prior studies have validated this rating scale against other scoring methods (5), and compared performance on the scale against both self-reported crashes as well as state records of motor-vehicle crashes (6, 7). In this scale, a score between 1 and 3 was incurred when a driver demonstrated multiple serious driving errors which reflected loss of the skill level required to complete the driving task safely in simple and complex traffic. Typically, in these cases, the DI was required to intervene on multiple occasions to prevent an accident or dangerous situation and, if undertaking a local licensing test, the driver's performance would result in a fail and possible loss of license. A score of 4 or 5 indicate poor driving and observation skills, while a score between 6 to 8 indicated average driving skills with some bad habits, and a score of 9 to 10 indicated excellent driving and observational skills. Drivers deemed as unsafe were counselled regarding their performance on the day and advised to follow up with their general practitioner. Inter-rater reliability of test scores between the OT and DI (using the same scale) was high (intra-class correlation = 0.94 (95% CI: 0.93–0.95), n = 548). The mean safety rating at the two sites were not statistically different (Canberra: Mean=5.95 (SD=1.57); Brisbane: Mean=5.91 (SD=2.07), Mean Difference = 0.032(-0.28,0.34), t(463.9)=0.20, p=0.84) and a small inter-site reliability test conducted at the Brisbane site confirmed the two OTs had comparable ratings of participant performance on the same route (intra-class correlation = 0.90 (95%CI:0.50,0.98), n=8 (OT1=5.25(1.28); OT2=5.63(1.19), Mean Difference = -0.38 (95%CI:-0.99, 0.25), t(7)=-1.42, p=0.20).

References.

1. Bertens D, Vos S, Kehoe P, Wolf H, Nobili F, Mendonça A, et al. Use of mild cognitive impairment and prodromal AD/MCI due to AD in clinical care: a European survey. Alzheimer's research & therapy. 2019;11(1):74.
2. Roberts J, Karlawish J, Uhlmann W, Petersen RC, Green R. Mild cognitive impairment in clinical care: a survey of American Academy of Neurology members. Neurology. 2010;75(5):425-31.
3. Eramudugolla R, Mortby ME, Sachdev P, Meslin C, Kumar R, Anstey KJ. Evaluation of a research diagnostic algorithm for DSM-5 neurocognitive disorders in a population-based cohort of older adults. Alzheimer's research & therapy. 2017;9(1):15.
4. Wood JM, Anstey KJ, Lacherez PF, Kerr GK, Mallon K, Lord SR. The on-road difficulties of older drivers and their relationship with self-reported motor vehicle crashes. J Am Geriatr Soc. 2009;57(11):2062-9.
5. Mallon K, Wood JM. Occupational therapy assessment of open-road driving performance: Validity of directed and self-directed navigational instructional components. American Journal of Occupational Therapy. 2004;58(3):279-86.

© 2020 Anstey KJ et al. *JAMA Network Open.*

6. Anstey KJ, Wood J, Caldwell H, Kerr G, Lord SR. Comparison of self-reported crashes, state crash records and an on-road driving assessment in a population-based sample of drivers aged 69-95 years. Traffic injury prevention. 2009;10(1):84-90.
7. Wood JM, Anstey KJ, Lacherez PF, Kerr GK, Mallon K, Lord SR. The On-Road Difficulties of Older Drivers and Their Relationship with Self-Reported Motor Vehicle Crashes. J Am Geriatr Soc. 2009;57(11):2062-9.

© 2020 Anstey KJ et al. *JAMA Network Open.*

eTable 1. STARD Checklist

Section & Topic	No	Item	Reported on page #
TITLE OR ABSTRACT			
	1	Identification as a study of diagnostic accuracy using at least one measure of accuracy (such as sensitivity, specificity, predictive values, or AUC)	1,3
ABSTRACT			
	2	Structured summary of study design, methods, results, and conclusions (for specific guidance, see STARD for Abstracts)	3
INTRODUCTION			
	3	Scientific and clinical background, including the intended use and clinical role of the index test	5
	4	Study objectives and hypotheses	5
METHODS			
Study design	5	Whether data collection was planned before the index test and reference standard were performed (prospective study) or after (retrospective study)	6
Participants	6	Eligibility criteria	6
	7	On what basis potentially eligible participants were identified (such as symptoms, results from previous tests, inclusion in registry)	6
	8	Where and when potentially eligible participants were identified (setting, location and dates)	6
	9	Whether participants formed a consecutive, random or convenience series	6
Test methods	10a	Index test, in sufficient detail to allow replication	7-8
	10b	Reference standard, in sufficient detail to allow replication	8, eMethods p3
	11	Rationale for choosing the reference standard (if alternatives exist)	5, eMethods p3
	12a	Definition of and rationale for test positivity cut-offs or result categories of the index test, distinguishing pre-specified from exploratory	7-8
	12b	Definition of and rationale for test positivity cut-offs or result categories of the reference standard, distinguishing pre-specified from exploratory	8, eMethods p3
	13a	Whether clinical information and reference standard results were available to the performers/readers of the index test	7
	13b	Whether clinical information and index test results were available to the assessors of the reference standard	7, 8
Analysis	14	Methods for estimating or comparing measures of diagnostic accuracy	9-10
	15	How indeterminate index test or reference standard results were handled	9-10
	16	How missing data on the index test and reference standard were handled	10

© 2020 Anstey KJ et al. *JAMA Network Open.*

		**17**	Any analyses of variability in diagnostic accuracy, distinguishing pre-specified from exploratory	12
		**18**	Intended sample size and how it was determined	6
	**RESULTS**			
	Participants	**19**	Flow of participants, using a diagram	30
		**20**	Baseline demographic and clinical characteristics of participants	24-27
		**21a**	Distribution of severity of disease in those with the target condition	24-27
		**21b**	Distribution of alternative diagnoses in those without the target condition	24-27
		**22**	Time interval between index test or clinical intervention and reference standard	8
	Test results	**23**	Cross tabulation of the index test results (or their distribution) by the results of the reference standard	24
		**24**	Estimates of diagnostic accuracy and their precision (such as 95% confidence intervals)	28-29
		**25**	Any adverse events from performing the index test or the reference standard	11
	**DISCUSSION**			
		**26**	Study limitations, including sources of potential bias, statistical uncertainty, and generalisability	14
		**27**	Implications for practice, including the intended use and clinical role of the index test	14-15
	**OTHER INFORMATION**			
		**28**	Registration number and name of registry	N/A
		**29**	Where the full study protocol can be accessed	N/A
		**30**	Sources of funding and other support; role of funders	16

© 2020 Anstey KJ et al. *JAMA Network Open.*

eTable 2. Criteria and Measures for Psychometric Classification of Mild Cognitive Impairment

**Criterion**	**Measure and cut-off scores**
1. No dementia	Mini-Mental State Exam (MMSE) > 23, and no known diagnosis
2. Subjective memory decline	Memory Complaints Questionnaire (MAC-Q) > 24 (1)
3. Objective cognitive impairment	1 standard deviation below age, gender and education adjusted z-score in at least 1 domain:
Complex Attention	Victoria Stroop Test – Time to complete Parts A and B (2)
Learning and Memory	California Verbal Learning Test (CVLT) Immediate Recall; Delayed Recall. (3)
Language	Controlled Oral Word Test (COWAT), Boston Naming Test (BNT-15) (4)
Perceptual-Motor	Benton Visual Retention Test (BVRT-Copy) (5)
Executive Function	Victoria Stroop Test Part C; Trail Making Test Part B; Wechsler Digit Span Backwards (6); Game of Dice Test (7)
4. Preserved basic ADLs/ minimal impairment in complex IADLs	Items adapted from Health and Retirement Survey – no reported difficulties due to cognition in shopping, meal preparation, using a map, making telephone calls, taking medications.

References:
(1) Crook TH, Feher EP, Larrabee GJ. Assessment of memory complaint in age-associated memory impairment: the MAC-Q. International psychogeriatrics. 1992;4(2):165-76.
(2) Strauss E, Sherman EM, Spreen O. A compendium of neuropsychological tests: Administration, norms, and commentary: American Chemical Society; 2006.
(3) Delis DC, Massman PJ, Kaplan E, Mckee R, Kramer JH, Gettman D. Alternate form of the California Verbal Learning Test: development and reliability. The Clinical Neuropsychologist. 1991;5(2):154-62.
(4) Mack WJ, Freed DM, Williams BW, Henderson VW. Boston Naming Test: shortened versions for use in Alzheimer's disease. Journal of Gerontology. 1992;47(3):P154-P8.
(5) Sivan AB. Benton visual retention test: Psychological Corporation San Antonio, TX; 1992.
(6) Wechsler D. Wechsler adult intelligence scale–Fourth Edition (WAIS–IV). San Antonio, TX: NCS Pearson. 2008;22:498.
(7) Brand M, Pawlikowski M, Labudda K, Laier C, von Rothkirch N, Markowitsch HJ. Do amnesic patients with Korsakoff's syndrome use feedback when making decisions under risky conditions? An experimental investigation with the Game of Dice Task with and without feedback. Brain and Cognition. 2009;69(2):279-90.

© 2020 Anstey KJ et al. *JAMA Network Open.*

eTable 3. Performance on Age, Sex, and Education Standardized Domain Measures of Neurocognitive Function for the Canberra Sample by Subgroup

Group	Attention	Memory	Language	Perceptual Motor	Executive Function
Comparison Group					
All	0.21 (-0.20,0.62)	0.34 (-0.33,0.88)	0.18 (-0.14,0.61)	0.22 (-0.13, 0.66)	0.16 (-0.17,0.52)
Cognitively Impaired Group					
MCI	-0.73 (-1.32,0.21)	-0.28 (-0.90,0.20)	-0.32 (-0.85,0.24)	-0.75 (-1.99,0.62)	-0.17 (-0.54,0.11)
DARS only	0.10 (-0.32, 0.50)	0.21 (-0.21,0.79)	0.03 (-0.38, 0.43)	0.11 (-0.31,0.66)	0.02 (-0.37,0.42)
Dementia	-1.29 (-2.24,-0.92)	-1.23 (-1.83,-0.71)	-1.40 (-2.01,-0.79)	-0.27 (-0.80,0.25)	-1.03 (-1.41,-0.65)
All	-0.41(-1.04,0.34)	-0.11(-0.71,0.32)	-0.22(-0.75,0.32)	-0.37(-1.28,0.66)	-0.13 (-0.56,0.29)

Note: normative data obtained from published sources; DARS – Driving Assessment and Rehabilitation Service, MCI – Mild Cognitive Impairment

© 2020 Anstey KJ et al. *JAMA Network Open.*

eTable 4. Multivariate Logistic Regression Coefficients for Combination of Factors Associated With On-Road Test Safety

	Complete case analysis (n=433)				Multiple imputation (n=559)			
Screening Measure	OR	95% CI	P-value	AUC (95% CI)	OR	95% CI	P-value	AUC
Multi-D	2.134	(1.565, 2.910)	<0.001	0.892 (0.849, 0.935)	1.979	(1.530, 2.559)	<0.001	0.870 (0.830, 0.911)
HPT	1.417	(1.114, 1.803)	0.005		1.284	(1.049, 1.571)	0.015	
UFOV	1.004	(1.002, 1.007)	0.001		1.004	(1.002, 1.006)	<0.001	

*Adjusted for data collection site. One individual has missing data for all covariates so was dropped from imputation.

© 2020 Anstey KJ et al. *JAMA Network Open.*

eTable 5. Logistic Regression

	On-Road Safety (Unsafe vs Safe)							
	Brisbane sample				Canberra sample			
	Vision impaired (n=124)		Comparison group (n=129)		Cognitively impaired (n=105)		Comparison group (n=202)	
Screening Measure	OR (95% CI)	P-value	OR (95% CI)	P-value	OR (95% CI)	P-value	OR (95% CI)	P-value
Useful Field of View (UFOV)	1.006 (1.002-1.009)	<0.001	1.009 (1.005-1.014)	<0.001	1.002 (0.998-1.006)	0.359	1.008 (1.004-1.012)	<0.001
DriveSafe	0.957 (0.934-0.980)	<0.001	0.939 (0.904-0.976)	0.002	0.957 (0.919-0.998)	0.039	0.937 (0.905-0.971)	<0.001
Maze Test	1.057 (1.019-1.097)	0.003	1.044 (1.003-1.088)	0.036	1.016 (0.996-1.037)	0.116	1.037 (1.003-1.072)	0.031
Trail Making Test B	1.011 (1.004-1.017)	0.001	1.009 (1.001-1.017)	0.033	1.008 (1.000-1.016)	0.042	1.016 (1.008-1.025)	<0.001
Multi-D Battery	2.196 (1.465-3.289)	<0.001	3.585 (1.734-7.412)	0.001	2.884 (1.416-5.873)	0.004	2.388 (1.278-4.463)	<0.001
Hazard Perception RT	1.746 (1.283-2.376)	<0.001	1.719 (1.138-2.596)	0.010	1.705 (1.171-2.484)	0.005	1.573 (1.113-2.224)	0.010
DriveSafe Intersection test	0.722 (0.502-1.038)	0.079	0.741 (0.443-1.238)	0.253	0.852 (0.591-1.228)	0.391	0.589 (0.428-0.812)	0.001
14-Item Road Law Test	0.964 (0.860-1.079)	0.476	0.786 (0.640-0.965)	0.073	0.918 (0.825-1.022)	0.119	0.899 (0.804-1.006)	0.063

© 2020 Anstey KJ et al. *JAMA Network Open.*

eTable 6. Sensitivity and Specificity Across All Subgroups

	Brisbane sample									
	Vision impaired (n=124)					Comparison group (n=129)				
Screening Measure	Sensitivity (%)	Specificity (%)	PPV (%)	NPV (%)	Correctly classified (%)	Sensitivity (%)	Specificity (%)	PPV (%)	NPV (%)	Correctly classified (%)
Useful Field of View (UFOV)	50.0	88.5	54.2	86.7	80.3	54.5	98.3	75.0	95.9	94.6
DriveSafe	57.7	81.2	45.4	87.6	76.2	72.7	74.6	21.0	96.7	74.4
Maze Test	96.1	45.8	32.5	97.8	56.6	63.6	61.0	13.2	94.7	61.2
Trail Making Test B	61.5	76.5	41.0	88.2	73.4	63.6	83.0	25.9	96.1	81.4
Multi-D Battery	75.0	75.8	45.0	92.0	75.6	80.0	78.2	25.0	97.7	78.3
Hazard Perception RT	73.1	61.5	33.9	89.4	63.9	45.4	84.7	21.7	94.3	81.4
DriveSafe Intersection test	43.5	76.1	32.3	83.7	69.4	28.6	79.6	8.0	94.7	76.7
14-Item Road Law Test	69.2	54.9	18.0	92.6	56.7	14.3	98.2	33.3	94.8	93.3
**Multivariate model**	**87.5**	**70.8**	**44.7**	**95.4**	**74.3**	**70.0**	**90.9**	**41.2**	**97.1**	**89.2**
	Canberra Sample									

© 2020 Anstey KJ et al. *JAMA Network Open.*

	Cognitively Impaired (n=105)					Comparison group (n=202)				
	Sensitivity (%)	Specificity (%)	PPV (%)	NPV (%)	Correctly classified (%)	Sensitivity (%)	Specificity (%)	PPV (%)	NPV (%)	Correctly classified (%)
Useful Field of View (UFOV)	75.0	55.3	19.2	94.0	57.7	93.7	55.9	16.1	99.0	59.1
DriveSafe	100.0	39.1	17.2	100.0	45.9	81.3	63.6	16.9	97.4	65.1
Maze Test	61.5	64.1	19.5	92.2	63.8	64.7	61.7	13.6	95.0	62.0
Trail Making Test B	83.3	55.1	20.0	96.1	58.4	50.0	87.2	25.8	95.2	84.2
Multi-D Battery	71.4	87.3	35.7	96.9	85.9	100.0	52.5	9.5	100.0	54.7
Hazard Perception RT	58.3	81.4	30.4	93.3	78.6	50.0	74.6	15.1	94.3	72.5
DriveSafe Intersection test	84.6	48.9	19.0	95.7	53.3	82.4	58.5	15.6	97.3	60.5
14-Item Road Law Test	69.2	54.9	18.0	92.6	56.7	47.1	74.6	14.8	93.7	77.2
**Multivariate model**	**83.3**	**91.8**	**50.0**	**98.3**	**91.0**	**83.3**	**80.3**	**16.7**	**99.0**	**80.1**

Note. Multivariate model includes HPT, Multi-D and UFOV.

© 2020 Anstey KJ et al. *JAMA Network Open.*
